# Epidemiology and molecular typing of multidrug-resistant bacteria in day care centres in Flanders, Belgium

**DOI:** 10.1017/S0950268823001528

**Published:** 2023-09-15

**Authors:** Stefanie van Kleef – van Koeveringe, Veerle Matheeussen, Hilde Jansens, Natascha Perales Selva, Dieter De Coninck, Katrien De Bruyne, Klaas Mensaert, Marjolein Kluytmans - van den Bergh, Jan Kluytmans, Herman Goossens, Wouter Dhaeze, on behalf of the i-4-1-Health Study Group

**Affiliations:** 1 Laboratory of Medical Microbiology, University Hospital Antwerp, Edegem, Belgium; 2 Department of Medical Microbiology, Vaccine & Infectious Disease Institute (VAXINFECTIO), University of Antwerp, Wilrijk, Belgium; 3 Department of Infection Control, University Hospital Antwerp, Edegem, Belgium; 4 bioMérieux, Augmented Diagnostics, Industrial Microbiology, Applied Maths NV, Sint-Martens-Latem, Belgium; 5 Department of Infection Control, Amphia Hospital, Breda, the Netherlands; 6 Julius Center for Health Sciences and Primary Care, UMC Utrecht, Utrecht University, Utrecht, the Netherlands; 7 Microvida Laboratory for Microbiology, Amphia Hospital, Breda, and Department of Medical Microbiology, University Medical Center Utrecht, Utrecht, The Netherlands; 8 Department Prevention, Agency for Care and Health, Leuven, Belgium

**Keywords:** MDRO, screening, day care centres, whole-genome sequencing, wgMLST, molecular typing, transmission

## Abstract

The global prevalence and spread of multidrug-resistant organisms (MDROs) represent an emerging public health threat. Day care centre (DCC) attendance is a risk factor for MDRO carriage in children and their environment. This study aimed to map the epidemiology of carriage and potential transmission of these organisms within 18 Flemish DDCs (Belgium). An MDRO prevalence survey was organised between November 2018 and February 2019 among children attending the centres. Selective chromogenic culture media were used for the detection of extended-spectrum beta-lactamase-producing *Enterobacterales* (ESBL-E), carbapenemase-producing *Enterobacterales* (CPE), and vancomycin-resistant Enterococci (VRE) in faecal swabs obtained from diapers or jars (n = 448). All isolated MDROs were subjected to resistance gene sequencing. A total of 71 of 448 samples (15.8%) yielded isolates of ESBL-E with a predominance of *Escherichia coli* (92.2% of ESBL-E) and ESBL resistance gene bla_CTX-M-15_ (50.7% of ESBL coding genes in *E. coli*). ESBL-E prevalence varied between DCCs, ranging from 0 to 50%. Transmission, based on the clonal relatedness of ESBL-E strains, was observed. CPE was identified in only one child carrying an *E. coli* with an OXA-244 gene. VRE was absent from all samples. The observed prevalence of ESBL-E in Flemish DCCs is high compared with previous studies, and our findings re-emphasise the need for rigorous hygiene measures within such centres to control the further spread of MDROs in the community.

## Introduction

The global prevalence and spread of multidrug-resistant organisms (MDROs) represent an emerging public health threat [1]. Day care centre (DCC) attendance is an important risk factor for MDRO carriage in children and their households [2], as it may facilitate the presence and spread of such organisms through the grouping of large numbers of children who have frequent close person-to-person contact and by the use of antibiotics [3–5].

While MDRO prevalence can be studied using conventional culture and identification techniques in combination with PCR to detect specific resistance genes, the study of their epidemiology requires in-depth molecular analysis to type strains and reveal clonal relatedness and possible transmission events [6, 7]. Analysis of the entire genome via whole-genome sequencing (WGS) allows discrimination of highly related lineages of bacteria and can map relevant genomic characteristics [8], [9]. In addition, whole-genome multilocus sequence typing (wgMLST), a gene-by-gene comparative approach that detects allelic variation at the genome level, can be used in outbreak settings [10].

A few studies have investigated the prevalence of MDRO carriage in European DCCs. The prevalence of faecal CTX-M-type ESBL-producing *Escherichia coli* isolates in French DCCs was reported to be 6.4% [11]. Likewise, in the Netherlands there is an overall prevalence of extended-spectrum cephalosporin-resistant (ESC-R) *E. coli* in DCC-attending children (<4 years old) of 4.5% and 8% in <1-year-old attendees [12]. In Belgium, children go to the DCCs until the age of 2.5 years, but due to the clusters of children <2.5 years, who are not potty trained, the MDRO transmission risk factors related to hygiene practices are high. Prevalence data on MDRO carriage in Belgian DCCs are scarce, and transmission of MDROs in this setting has not been studied. We therefore explored the prevalence and transmission of faecal carriage of ESBL-E, carbapenem-resistant *Enterobacterales* (CPE), and vancomycin-resistant enterococci (VRE) in 18 Flemish DCCs using conventional culture techniques and wgMLST.

## Materials and methods

### Study design

A cross-sectional prevalence survey, the i-4-1-Health project [13], was organised in the cross-border region of the southern part of the Netherlands and Flanders, Belgium. The prevalence of MDROs was studied in livestock farms, tertiary care hospitals, nursing homes, and DCCs. This study presents the results from 18 Flemish DCCs, obtained between November 2018 and February 2019.

### Sampling and microbiological analysis

The methodology used in the i-4-1-Health project is described by Kluytmans-van den Bergh et al. [13]. Briefly, faeces present in diapers or collection jars from DCC children were sampled with a FecalSwab (Copan, Brescia, Italy), stored at 2–8 °C, and processed within 48 hours after collection. Cultures and identification analyses were performed at the central hospital microbiology laboratory. Swabs were pre-enriched in a non-selective tryptic soy broth (TSB) (Copan, Brescia, Italy) and directly cultured on blood agar plates. After 18–24 h of incubation, the enrichment broths were subcultured on selective chromogenic (ChromID®) agars, namely ESBL, CARBA, OXA-48, and VRE (bioMérieux, Marcy-l’Étoile, France). After 18–24 h of incubation of the ESBL, CARBA, and OXA-48 plates and 48 h of incubation of VRE plates, MALDI-TOF MS (Bruker, Billerica, USA) was used for species identification. All isolates were tested for antibiotic susceptibility by disc diffusion assays, and the results were interpreted with reference to EUCAST clinical breakpoints (v8.1) [14]. ESBL production was confirmed by the inhibition of β-lactamase activity by clavulanic acid [15]. The identification of CPE-like phenotypes was based on the screening cut-off values of meropenem and temocillin and confirmed by whole-genome sequencing (WGS). Vancomycin resistance was phenotypically confirmed by vancomycin and teicoplanin ETEST® (bioMérieux) according to EUCAST with a MIC of ≥8 mg/L. All isolates were stored at −80 °C.

### Whole-genome sequencing

MH broths were inoculated with phenotypically confirmed ESBL-E, CPE, and VRE isolates and cultured in Muller–Hinton (MH) broth (BD, Erembodegem, Belgium) for 18 to 24 h. DNA was isolated from 500 μl of the broth culture using the MasterPure™ Complete DNA and RNA Purification Kit (Epicenter, Madison, USA). DNA concentration was measured with a Qubit Fluorimeter 2.0 (ThermoFisher Scientific, Waltham, USA) using the Qubit Double-Stranded DNA (dsDNA) HS Assay Kit (Life Technologies, Carlsbad, USA). A concentration of 0.24–0.30 ng/μl of bacterial DNA was used for library preparation using the Nextera XT Library Preparation Kit with the Nextera XT v2 Index Kit (Illumina, San Diego, USA), according to the manufacturer’s instructions. Sequencing of the library was performed on a MiSeq sequencer, using the MiSeq Reagent Kit v2 generating 250-bp paired-end reads. A harmonisation study for WGS was performed within the i-4-1-Health project [16].

### Species confirmation

For each isolate, the MALDI-TOF MS identification was compared to the species prediction via sequencing [17].

### wgMLST analysis

wgMLST analysis was performed using BioNumerics software v7.6.3. (Applied Maths, bioMérieux, Belgium). To determine the allele number(s) corresponding to a unique allele sequence for each locus present in the genome of a strain, two different algorithms were performed: The assembly-free (AF) allele calling uses a k-mer approach starting from the raw sequence reads and the assembly-based (AB) allele calling uses a BLAST-based search with assembled genomes. The results of both algorithms were combined into a single set of allele assignments or consensus calls. Only the genes with valid start/stop codons, no ambiguous bases or internal stop codons, were assigned an allele number. Based on the consensus allelic profiles, a similarity matrix was calculated, using normalisation for missing values. This similarity matrix served as the basis for UPGMA clustering. The definition of loci and alleles was captured in a wgMLST scheme created by Applied Maths NV (bioMérieux) using publicly available genome data sets. The wgMLST scheme for *Klebsiella pneumoniae* consisted of 19.086 loci, *E. coli* 14.836 loci, *Enterobacter cloacae* 15.605 loci, *Enterococcus faecium* 5.489 loci, and *Enterococcus faecalis* 5.285 loci.

### Clonal relatedness

Clonal relatedness between isolates was determined based on the similarity of wgMLST allelic profiles. Similarity thresholds were determined by combining sequencing and epidemiological data from well-described bacterial outbreaks in different countries of varying duration and involved different sequence types. These highly variable data gave no clear-cut thresholds by which to separate or outbreak from sporadic strains. Therefore, an upper and lower threshold was defined (Table S1 of the Supplementary Material). If the allelic profiles of two strains showed similarity above the upper threshold, these strains were considered clonally related. If similarity was below the lower threshold, the strains were considered not clonally related. Likewise, for similarity values falling between these thresholds, no conclusion regarding clonal relatedness could be made based only on the wgMLST allelic profiles. In such cases, the epidemiological data were used to support an informed decision.

### Antibacterial resistance prediction

Using a BLAST-based approach requiring at least 95% identity with the reference sequence and at least 95% reference length coverage, genes known to confer resistance were identified from the assembled genomes. Resistance mediated by point mutations in specific genes was also identified by the same approach. Reference sequences and mutations from the Center for Genomic Epidemiology’s ResFinder (database version 2019-08-21) and PointFinder (database version 2019-07-02) databases, respectively, were used.

### Statistical analysis

The analysis of MDRO prevalence was primarily descriptive and presented as an absolute number, percentage, and mean for individual DCCs and overall. ANOVA was used to test statistical differences in MDRO prevalence between individual sites. All analyses were performed with IBM SPSS Statistics version 22.0 (IBM Corp., Armonk, New York, USA).

### Ethical considerations

The study protocol was reviewed and approved by the Ethics Committee of the University Hospitals Leuven (Leuven, Belgium) (S61807). The study was judged to be beyond the scope of the law on experiments on humans dated 7 May 2004. Written informed consent for faecal sampling for microbiological analysis was obtained from the legal representatives of all participants.

## Results

### Sample numbers and microbial species

In total, 18 DCCs participated in the i-4-1-Health project in Flanders, where, on average, six groups per centre were tested. Four hundred and forty-eight swabs from diapers or collection jars were obtained for MDRO screening; no swabs were excluded due to poor sampling quality. The total number of children screened in a DCC ranged from 6 to 56, with a mean overall participation rate of 40.0% (range 18.3% to 58.3%) (Table 1). The mean prevalence of ESBL-E was 15.8%, with significant variations observed among different centers. This ranged from 0% in four centers (6, 13, 15, and 16) to 50.0% in centre 4 (*p* < 0.001). Seventy-seven isolates were recovered from 71 ESBL-E-positive samples. *E. coli* was predominant (n = 71; 92.2%) and other species isolates were *Citrobacter freundii* (n = 2), and one representative each of *Citrobacter farmeri*, *Klebsiella aerogenes*, *K. pneumoniae*, and *E. cloacae.* Co-carriage of multiple ESBL isolates was detected in six children: four with two different *E. coli*, one with *E. coli* and *C. freundii,* and another with *E. coli*, *K. pneumoniae,* and *K. aerogenes.* One CPE isolate was detected in DCC 18 on the ESBL medium with genotype OXA-244 but was not detected on the OXA-48 or CARBA plates. No VRE were isolated.

**Table 1.** Participation rate and MDRO presence in swabs from day care centre (DCC) attending children

	% swabbed children	N samples	ESBL-E n (%)
DCC 1	43.6	44	2 (4.5)
DCC 2	57.1	56	7 (12.5)
DCC 3	34.3	34	12 (35.3)
DCC 4	25.0	14	7 (50.0)
DCC 5	18.3	11	1 (9.1)
DCC 6	20.0	15	0 (0.0)
DCC 7	46.9	23	2 (8.7)
DCC 8	28.3	32	5 (15.6)
DCC 9	58.3	28	8 (28.6)
DCC 10	50.0	28	2 (7.1)
DCC 11	43.5	40	15 (37.5)
DCC 12	37.2	16	2 (12.5)
DCC 13	34.1	14	0 (0.0)
DCC 14	51.1	24	2 (8.3)
DCC 15	31.6	18	0 (0.0)
DCC 16	20.0	6	0 (0.0)
DCC 17	44.4	24	2 (8.3)
DCC 18	21.4	21	3 (14.2)
Total DCCs	40.0	448	71 (15.8)

Abbreviations: DCC, day care centre; ESBL-E, extended-spectrum beta-lactamase-producing *Enterobacterales.*

### Antimicrobial susceptibility

**Figure 1.**
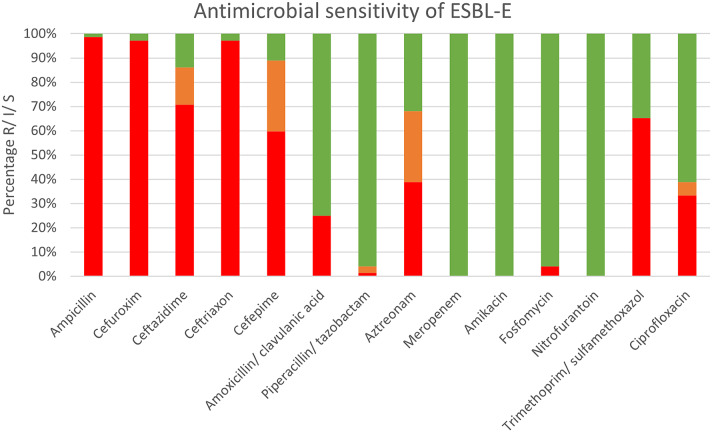
Antimicrobial resistance profile of 71 fecal ESBL E. coli isolated from children in DCCs. Isolates were tested for antibiotic resistance against 14 clinically relevant antibiotics using disk diffusion. The figure shows the resistant isolates in red, the susceptible ones in green and the intermediate susceptible ones in orange.

The antibiotic resistance profiles of the ESBL-producing *E. coli* isolates are depicted in Figure 1. Almost all (98.6%) showed resistance to ampicillin, with high levels of resistance to cephalosporins. One-quarter of the isolates were resistant to the amoxicillin/clavulanic acid combination, whereas 1.4% were resistant to piperacillin/tazobactam. In addition, monobactam and aztreonam showed relatively high rates, with 38.9% resistant and 29.2% intermediate susceptible isolates. All ESBL *E. coli* isolates were susceptible to meropenem and amikacin. Resistance rates to fosfomycin, nitrofurantoin, trimethoprim/sulfamethoxazole, and ciprofloxacin were 4.2%, 0%, 65.3%, and 33.3%, respectively.

Phenotypic resistance of all ESBL *E. coli* isolates was confirmed by the presence of a resistance gene with the following distribution: bla_CTX-M-15_ (n = 36; 50.7%), bla_CTX-M-14b_ (n = 14; 19.7%), and bla_CTX-M-27_ (n = 14; 19.7%). Other beta-lactamase resistance genes were bla_CTX-M-14_ (n = 2) and one each of bla_CTX-M-1_, bla_CTX-M-3_, bla_CTX-M-55_, bla_CTX-M-203_, and bla_SHV-12_.

**Figure 2.**
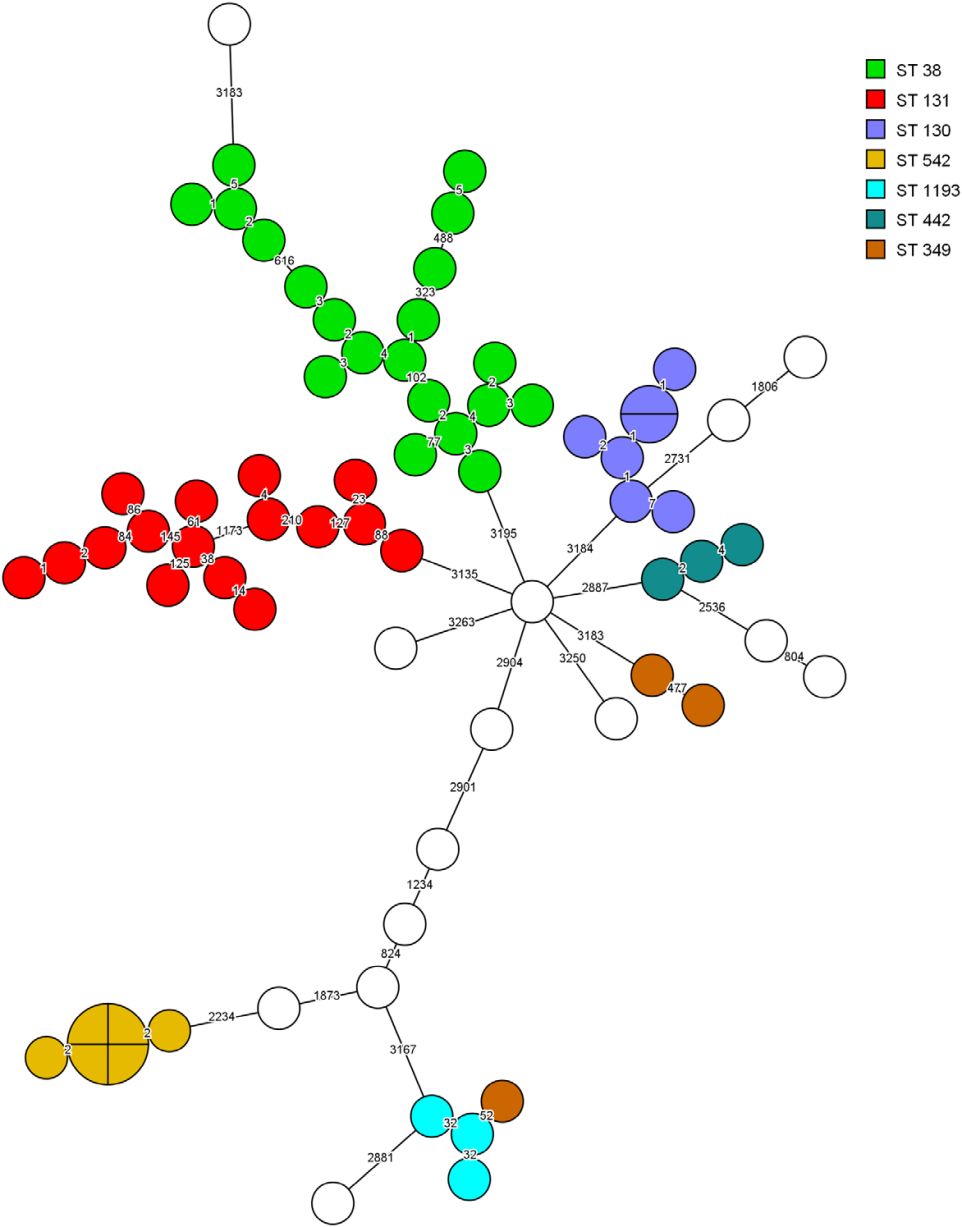
Minimum spanning trees of Escherichia coli ESBL isolates (n=71) based on wgMLST analysis. Isolates are represented by circles connected by branches proportional to the allelic distance. Colors represent the ST type. The white color represent less common ST types (<= 2 isolates).

**Figure 3.**
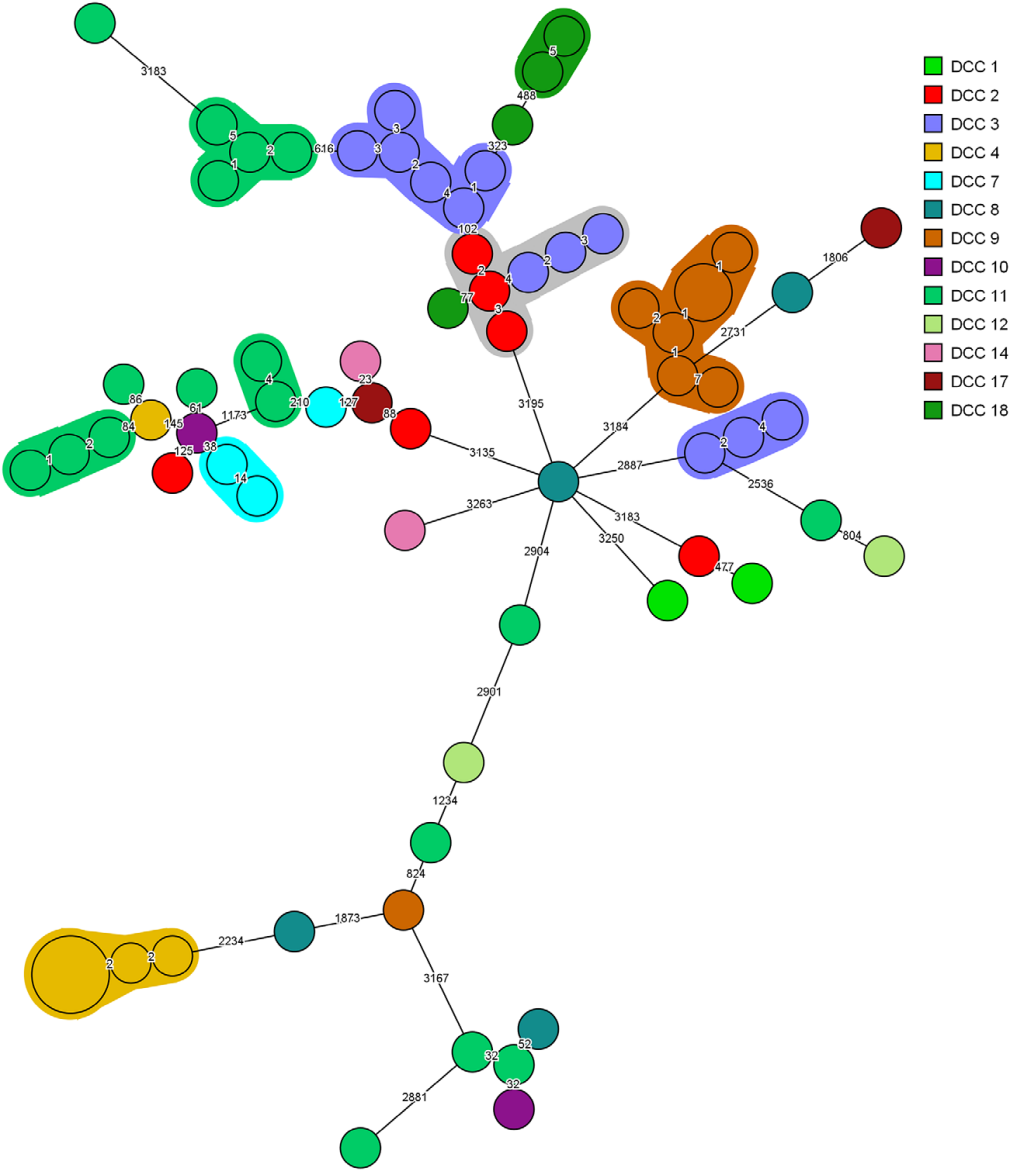
Minimum spanning trees of ESBL-producing Escherichia coli isolates (n = 71) based on wgMLST analysis. Isolates are represented by circles connected by branches proportional to the allelic distance. Colors represent the origin locations, the shading represents clusters.

Minimum spanning trees, colour-coded, based on ST type in Figure 2, and DCC origin in Figure 3 illustrate the clonal relatedness of wgMLST allelic profiles among the 71 ESBL *E. coli* isolates. ST 38 was the predominant MLST type (n = 20; 28.2%), followed by ST 131 (n = 16; 22.5%) and ST 130 (n = 7; 9.9%). Transmission of individual clones was observed in seven centres (Figure 3). Ten clonal clusters were evident, of which four comprised over five isolates. Three of the latter clusters consisted of isolates recovered from the same DCC, that is six each from centres 3 and 4 and seven from centre 9. Likewise, a cluster of six ST38 isolates was identified in DCC 2 and DCC 3, and in DCC 11, three small, unrelated clusters were detected.

## Discussion

In this cross-sectional survey, the prevalence of ESBL-E in Flemish DCCs was 15.8%. Compared to reported MDRO prevalence in similar settings in other countries (6.4% CTX-M-producing *E. coli* in France [11] and 8% ESC-R in the Netherlands [12]), the overall prevalence in Flanders is remarkably high. However, we found the ESBL-E prevalence in the latter region to vary markedly as this strain phenotype was not detected in four of the 18 centres surveyed. Further research on the different hygiene measures in the DCCs might clarify the difference in their prevalence. Moreover, clonal transmission of strains was evident within some study centres.

Compared to previous research in Flemish hospitals and nursing homes, it is remarkable that *E. coli* ST 38 was the most common ESBL-E type in the study cohort. This sequence type is known to be abundant in livestock, particularly poultry, and has been identified along the food production chain [18, 19], but it has predominantly been associated with urinary tract infections [20]. The spread of *E. coli* ST 38 within households that might be facilitated through attendance at DCCs is a significant risk factor for the carriage of ESBL-producing bacteria in children and their parents [2]. In contrast to the occurrence of *E. coli* ST38 in DCCs, the predominance of bla_CTX-M-15_ in this study is in line with the global human epidemiology of ESBL-producing bacteria [21].

Only one CPE-producing *E. coli* with an OXA-244 gene was detected on ChromID ESBL agar. OXA-244-producing *E. coli* isolates can pose a challenge for clinical laboratories as they may fail to grow on carbapenem-selective media or may not be detected by carbapenemase-specific tests [22]. OXA-244 is a single-point mutant derivative of OXA-48 with reduced carbapenemase activity. An increase in this genotype among *E. coli* isolates has been observed in different countries within the EU [23], including Germany [24], Switzerland [25], the Netherlands, Spain, the United Kingdom, and France [26]. Interestingly, in this case, the OXA-244-producing isolates were mostly of ST 38 [23].

Transmission of clonal-related ESBL *E. coli* was observed in seven DCCs, where high ESBL-E prevalence was detected. Nevertheless, the criteria applied for the allocation of an isolate to a specific clone are challenging. First, the commonly used thresholds are based on outbreak data from different countries and within a broad timespan, making them widely applicable, but not area- or time-specific. Our data were generated within a relatively small region, Flanders, and comparisons within a limited timeframe might require the use of more narrow thresholds. However, because the data were collected within the i-4-1-Health project, which included different countries, fixed thresholds were applied throughout all project data for uniformity reasons. Second, the thresholds are method- and analysis programme–specific and cannot be transferred directly to other methodologies [27]. Consequently, no apparent universally applicable thresholds for clonal definitions in the day care setting are available and warrant further exploration.

To conclude, our study shows that the overall prevalence of ESBL-E in Flemish DCCs is remarkably high compared with previous studies in neighbouring countries. However, the prevalence of this resistance group varies significantly according to their origins. ESBL *E. coli* isolates harbouring blaCTX_-M-15_ were predominant in our centres, with ST38 being the most frequent genotype. In the sites with high ESBL-E prevalence, the transmission of individual strains was observed. These results therefore re-emphasise the necessity for further research on hygiene measures and practices in DCCs to further inform training and awareness within centres to reduce the spread of MDROs in this setting and the wider community.

## Supporting information

van Kleef – van Koeveringe et al. supplementary material van Kleef – van Koeveringe et al. supplementary material

## Data Availability

The data that support the findings of this study are available from the corresponding author, Stefanie van Kleef-van Koeveringe, upon reasonable request.

## Supplementary material

For supplementary material accompanying this paper visit http://doi.org/10.1017/S0950268823001528. click here to view supplementary material

## I-4-1-health study group

The i-4-1-Health study group includes Lieke van Alphen (Maastricht University Medical Center+, Maastricht, the Netherlands), Nicole van den Braak (Avans University of Applied Sciences, Breda, the Netherlands), Caroline Broucke (Agency for Care and Health, Brussels, Belgium), Anton Buiting (Elisabeth-TweeSteden Ziekenhuis, Tilburg, the Netherlands), Liselotte Coorevits (Ghent University Hospital, Ghent, Belgium), Sara Dequeker (Agency for Care and Health, Brussels, Belgium and Sciensano, Brussels, Belgium), Jeroen Dewulf (Ghent University, Ghent, Belgium), Wouter Dhaeze (Agency for Care and Health, Brussels, Belgium), Bram Diederen (ZorgSaam Hospital, Terneuzen, the Netherlands), Helen Ewalts (Regional Public Health Service Hart voor Brabant, Tilburg, the Netherlands), Herman Goossens (University of Antwerp, Antwerpen, Belgium and Antwerp University Hospital, Antwerp, Belgium), Inge Gyssens (Hasselt University, Hasselt, Belgium), Casper den Heijer (Regional Public Health Service Zuid-Limburg, Heerlen, the Netherlands), Christian Hoebe (Maastricht University Medical Center+, Maastricht, the Netherlands, and Regional Public Health Service Zuid-Limburg, Heerlen, the Netherlands), Casper Jamin (Maastricht University Medical Center+, Maastricht, the Netherlands), Patricia Jansingh (Regional Public Health Service Limburg Noord, Venlo, the Netherlands), Jan Kluytmans (Amphia Hospital, Breda, the Netherlands, and University Medical Center Utrecht, Utrecht University, Utrecht, the Netherlands), Marjolein Kluytmans-van den Bergh (Amphia Hospital, Breda, the Netherlands, and University Medical Center Utrecht, Utrecht University, Utrecht, the Netherlands), Stefanie van Kleef-van Koeveringe (Antwerp University Hospital, Antwerp, Belgium), Sien de Koster (University of Antwerp, Antwerp, Belgium), Christine Lammens (University of Antwerp, Antwerp, Belgium), Isabel Leroux-Roels (Ghent University Hospital, Ghent, Belgium), Hanna Masson (Agency for Care and Health, Brussel, Belgium), Ellen Nieuwkoop (Elisabeth-TweeSteden Ziekenhuis, Tilburg, the Netherlands), Anita van Oosten (Admiraal de Ruyter Hospital, Goes, the Netherlands), Natascha Perales Selva (Antwerp University Hospital, Antwerp, Belgium), Merel Postma (Ghent University, Ghent, Belgium), Stijn Raven (Regional Public Health Service West-Brabant, Breda, the Netherlands), Veroniek Saegeman (University Hospitals Leuven, Leuven, Belgium), Paul Savelkoul (Maastricht University Medical Center+, Maastricht, the Netherlands), Annette Schuermans (University Hospitals Leuven, Leuven, Belgium), Nathalie Sleeckx (Experimental Poultry Centre, Geel, Belgium), Arjan Stegeman (Utrecht University, Utrecht, the Netherlands), Tijs Tobias (Utrecht University, Utrecht, the Netherlands), Paulien Tolsma (Regional Public Health Service Brabant Zuid-Oost, Eindhoven, the Netherlands), Jacobien Veenemans (Admiraal De Ruyter Hospital, Goes, the Netherlands), Dewi van der Vegt (PAMM Laboratory for Pathology and Medical Microbiology, Veldhoven, the Netherlands), Martine Verelst (University Hospitals Leuven, Leuven, Belgium), Carlo Verhulst (Amphia Hospital, Breda, the Netherlands), Pascal De Waegemaeker (Ghent University Hospital, Ghent, Belgium), Veronica Weterings (Amphia Hospital, Breda, the Netherlands), Clementine Wijkmans (Regional Public Health Service Hart voor Brabant, Tilburg, the Netherlands), Patricia Willemse-Smits (Elkerliek Hospital, Helmond, the Netherlands), and Ina Willemsen (Amphia Hospital, Breda, the Netherlands).
