# Supplementary material for: Epidemiology and molecular typing of multidrug-resistant bacteria in day care centres in Flanders, Belgium
Source: Epidemiol Infect. 2023 Sep 15;151:e156. doi: 10.1017/S0950268823001528 (PMC10548538; doi:10.1017/S0950268823001528)
Supplement: van Kleef – van Koeveringe et al. supplementary material [file S0950268823001528sup001.docx]

*Table S1. Thresholds used to determine clonal relatedness based on the similarity of the allelic profiles of two strains.*

|  | Similarity (%) threshold | |
| --- | --- | --- |
| Organism | No relatedness | Relatedness |
| *Citrobacter spp* | <90.00 | >98.00 |
| *Enterobacter cloacae* | <98.40 | >99.43 |
| *Enterococcus faecalis* | <98.00 | >99.82 |
| *Enterococcus faecium* | <92.40 | >93.70 |
| *Escherichia coli* | <90.12 | >97.54 |
| *Klebsiella aerogenes* | <95.10 | >97.70 |
| *Klebsiella oxytoca* | <90.00 | >99.13 |
| *Klebsiella pneumoniae* | <96.12 | >99.64 |
